# Pneumoretroperitoneum with subcutaneous emphysema after a post colonoscopy colonic perforation

**DOI:** 10.1016/j.ijscr.2019.03.030

**Published:** 2019-04-16

**Authors:** Sahned Jaafar, Suy Sen Hung Fong, Abdul Waheed, Subhasis Misra, Keyur Chavda

**Affiliations:** Brandon Regional Hospital, Brandon, FL, 33511, USA

**Keywords:** Intraperitoneal perforation, Retroperitoneal perforation, Colonic perforation, Colonoscopy

## Abstract

**Introduction:**

Colonoscopy is considered as one of the most common performed procedures for both diagnostic and therapeutic purposes. Serious complication after colonoscopy could occurs like bleeding or perforation. Majority of the perforations are intraperitoneal, while extraperitoneal perforations are very rare and it may cause pneumoretroperitoneum, pneumomediastinum, pneumothorax and subcutaneous emphysema.

**Presentation of the case:**

A case report was described of 80-year-old female who underwent a diagnostic colonoscopy for recurrent abdominal pain who presented with severe abdominal pain and tenderness with CT findings of pneumoperitoneum, pneumoretroperitoneum and subcutaneous emphysema. The patient successfully treated surgically with laparoscopic Hartman procedure.

**Discussion:**

Colonic perforation is rare complication of colonoscopy that can manifest intraperitoneal, extraperitoneal or a combination of both. Patient risk factors include advance age, female sex, diverticulosis, previous abdominal surgery and pre-existing colonic strictures. Technical risk factors includes excessive shearing forces during endoscopic insertion, dilation, biopsy and using electrocautery. Majority of the intraperitoneal perforation warrant a surgical intervention whereas isolated extraperitoneal perforation may be managed conservatively.

**Conclusion:**

Combined intraperitoneal and extraperitoneal colonic perforation are rare presentations following diagnostic colonoscopy and often difficult to diagnose based on the clinical manifestation only. Understanding the manifestation of extraperitoneal perforation will help to properly identify the condition and preventing morbidity and mortality in these patients.

## Highlights

• Colonic perforation after colonoscopy could be intraperitoneal, extraperitoneal or a combination of both.
• Majority of the perforations are intraperitoneal.
• Risk factors include advance age, female sex, diverticulosis, previous abdominal surgery and colonic strictures.
• Extraperitoneal perforations can manifest as pneumoretroperitoneum, pneumomediastinum, pneumothorax and/or subcutaneous emphysema.
• Non operative management in isolated retroperitoneum while surgery required in majority of peritoneal perforation.

## 1 Introduction

Colonoscopy is a commonly performed diagnostic and therapeutic procedure for colorectal disorders [[1], [2], [3]]. The frequencies of colonoscopies performed annually in the United States (US) are increasing, and in 2012 alone more than 15 million colonoscopies were performed [4]. A properly performed colonoscopy is generally safe and well-tolerated; however, complications following colonoscopy are not uncommon [1]. Although rare, the colonoscopy may lead to life-threatening conditions, with colonic perforations (CP) being the fatal one with an overall incidence rate of 0.03–8% [5]. There has been convincing evidence that therapeutic colonoscopies are associated with significantly higher CP than diagnostic colonoscopies [5,6]. Lahsirwat et al. reported a perforation rate of 0.016−0.2% following diagnostic colonoscopy, and 5% following therapeutic colonoscopy [5,6].

Furthermore, the majority of the CP are IPP, while RPP are extremely rare [7]. An IPP typically presents with abdominal pain, distension and peritonitis, while RPP presents with the atypical findings including subcutaneous crepitus, shortness of breath (SOB), chest pain, and neck swelling [5]. Moreover, CT abdomen is the best diagnostic study to differentiate between IPP and RPP, with specific findings such as pneumo-retroperitoneum and possible pneumomediastinum, the air in the neck, and groin through the fascial plane and along the course of blood vessels favoring the RPP [5]. Surprisingly, to the best of our knowledge, only 20 cases with concomitant IPP and RPP have been reported in the literature so far [5]. The current case report has been reported in line with the SCARE criteria [8], is a valuable addition to the limited available literature on this rare condition.

## 2 Case report

**Fig. 1:**
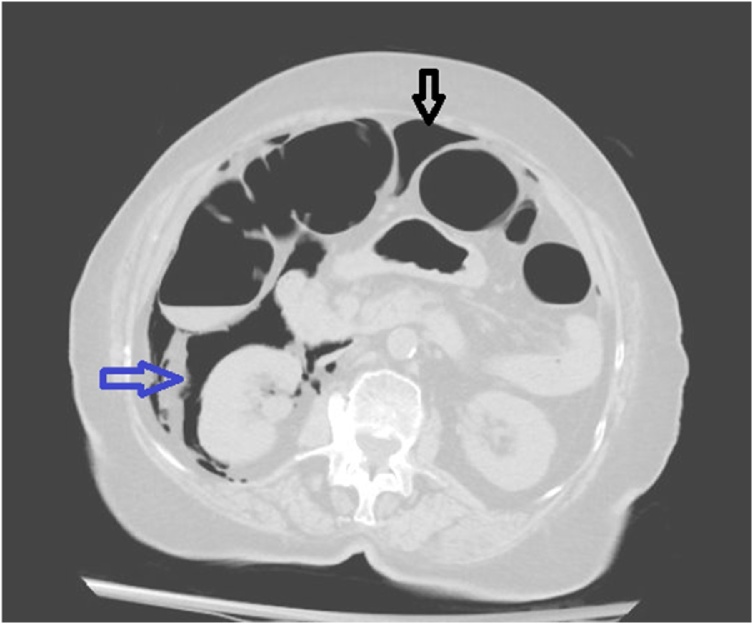
Intraperitoneal (IPP) and retroperitoneal (RPP) presence of air following colonoscopy. Coronal plane CT scans abdomen immediately following colonoscopy confirms massive intraperitoneal (subdiaphragmatic) air (Black arrow), and retroperitoneal area (Blue air).

**Fig. 2:**
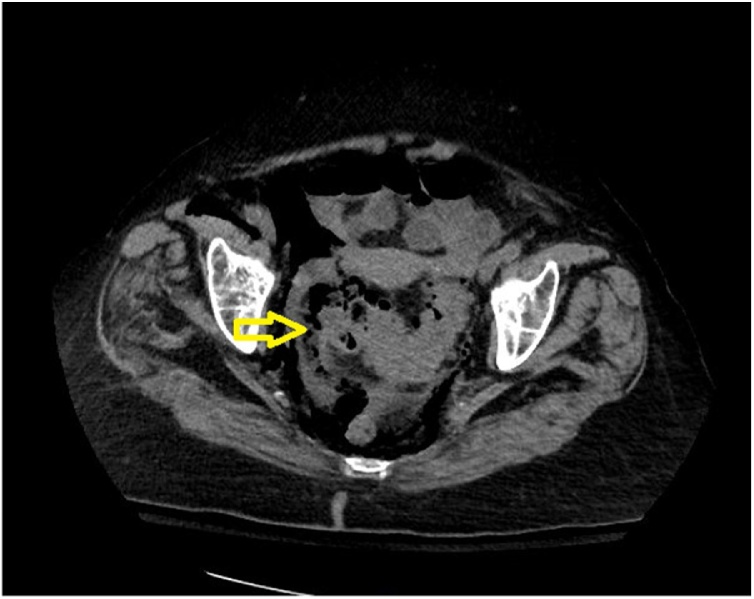
Thickened rectosigmoid with surrounding air. Coronal plane CT scans abdomen immediately following colonoscopy indicating thickened rectosigmoid with surrounding air (Yellow arrow).

**Fig. 3:**
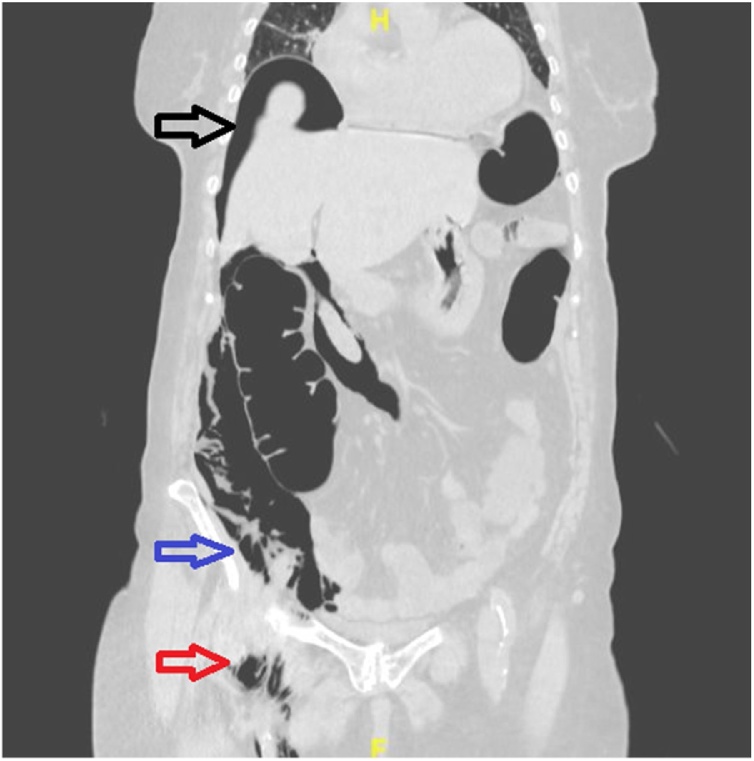
Intraperitoneal (IPP) and retroperitoneal (RPP) presence of air following colonoscopy. Sagittal view CT scans abdomen immediately following colonoscopy confirms massive intraperitoneal (subdiaphragmatic) air (Black arrow), retroperitoneal area (Blue air), and right groin air (Red arrow).

An 80-year old Hispanic American female patient presented for the evaluation of recurrent abdominal pain, and excessive bloating after every meal, without any significant past history of weight loss and change in bowel habits. At initial presentation, her vital signs were within normal range. CT abdomen was insignificant. At this point, she was scheduled for the esophagogastroduodenoscopy (EGD), which showed no significant abnormality. However, her symptoms persisted, and one week later she was scheduled for colonoscopy by a senior gastroenterologist for the evaluation of lower gastrointestinal tract. She was vitally stable, and colonoscopy was passed through the anal canal under the direct visualization. Shortly after the introduction of the scope, significant diverticulosis and kinking were seen the level of the sigmoid colon. Every effort was made to pass, the scope safely beyond that level, but it was not successful; the procedure caused significant discomfort requiring higher dose of propofol and midazolam and the decision made not to proceed further. The scope was withdrawn, and the patient returned to the recovery room. Two hours following colonoscopy, the patient complained of severe LLQ abdominal pain, but she was vitally stable. Additionally, her abdomen was soft; however, significant tenderness in the LLQ of abdomen and subcutaneous crepitus in the right upper thigh was noticed. She was immediately rushed for CT abdomen which revealed massive pneumoperitoneum, pneumo-retroperitoneum, and subcutaneous emphysema (Fig. 1, Fig. 2, Fig. 3).

At this point, the decision was made to take her back to the operating room (OR). She received proper preoperative intravenous fluids and broad-spectrum antibiotics. Distal sigmoid colon perforation with feculent peritonitis was seen, and laparoscopic Hartman procedure was performed. The postoperative period was uneventful, and her condition improved gradually. Her stoma was functioning very well, and diet advanced gradually. She was discharged home with home health care and scheduled for postoperative follow up in the clinic.

## 3 Discussion

Since 1970, colonoscopy has been widely adopted by the physicians across the world as the primary diagnostic tool for patients with suspected bowel lesions [9]. Colonoscopy is a useful diagnostic and therapeutic outpatient procedure for the colorectal benign and neoplastic lesions in the US [2,3]. Due to its utmost importance for colorectal cancer screening, centers for disease control and prevention (CDC) has predicted that by 2024, more than 80% of the US population between the ages of 50–75 years will receive screening colonoscopies for colorectal cancer [10].

Although colonoscopy is considered an extremely safe procedure, serious complications can occur following colonoscopy, and iatrogenic CP being the most lethal ones [11]. Additionally, there is no standard agreement regarding the incidence of CP post colonoscopy, Chen et al. in a recent study of long-term follow up 29 patients of CP following colonoscopy reported the incidence of perforation could range from 0.04% for diagnostic colonoscopies, and 0.7% for therapeutic colonoscopies [9].

Interestingly, in a prospective analysis of 13,580 colonoscopies, Wexner et al. proposed that the complication rates following colonoscopies are associated neither with the experience level nor with the number of the colonoscopies carried out annually [12,13]. However, certain risk factors including old age, diverticulosis, previous abdominal surgery, inflammatory bowel disease (IBD), colonic stricture, and therapeutic colonic interventions can contribute significantly to CP following colonoscopy [11]. Also, the mechanism of CP following colonoscopy has been widely debated in the available literature so far. It has been suggested that perforations may results from either excessive air insufflation (pneumatic perforations), excessive pressure by the colonoscope (mechanical perforations), or after performing improper therapeutic colonoscopies (polypectomy, electrocautery injury) [11,13,14].

Although CP can occur at any intestinal locations, the most commonly affected regions are sigmoid colon followed by the caecum [15,16]. In a study describing the rate of perforation at various locations, Iqbal et al. described that rectosigmoid junction and the sigmoid colon are most commonly effected areas (52%), followed by cecum (17%), ascending colon (14%), transverse colon (7%), descending colon (8%), and rectum (1%) [17]. Additionally, most of the perforation mentioned above presents predominantly as IPP, while the RPP and combined types of perforations are scarce [15]. In pure isolated RPP, subcutaneous emphysema is usually the first and most common clinical manifestation as it offers the least resistance to expansion [5]. Also, following the initial perforation in RPP, the air may pass into mediastinum, neck or groin by either dissection along the facial planes, or along with the blood vessels and psoas muscle [5].

Advances in radiological imaging have also permitted better delineation and improved sensitivity in detecting CP following colonoscopy. Cho et al. in a study of perforations caused by diagnostic endoscopy and therapeutic colonoscopic interventions reported that abdominal roentgenogram (AR) is a cost-effective and useful method in detecting subdiaphragmatic free air (SFA), with a positive predictive value of 92% [18]. However, sometimes SFA cannot be detected by AR. In this setting, CT abdomen offers great help in detecting the presence of not only SFA but also micro-perforations and abscess [17].

Treatment of CP has historically been either conservative management or surgical intervention depending on the type of CP. Tiwari et al. reported that 75% of isolated RPP were treated conservatively, while 60% with combined IPP and RPP need surgical intervention [5]. However, occasionally the combined perforation can be managed non operatively if the patient is hemodynamically stable with a benign abdominal exam [5]. Up to 85% of IPP need surgical intervention, and primary repair could be achieved in 68% of the cases while approximately 32% need stoma [19]. The remaining 15% of the patients can be successfully treated non-operatively or can be managed with endoscopic clip closure if the perforation is small and recognized at the time of the procedure [19].

## 4 Conclusion

Combined IPP and RPP are rare presentations following diagnostic colonoscopy and often difficult to diagnose based on the clinical manifestation only. Clinically deteriorating patients following colonoscopy should alert the physicians to possible CP. CT abdomen provides excellent diagnostic information in CP patients, which can be managed either conservatively or surgically depending on the location and type of the CP.

## Conflicts of interest

Nothing to disclose.

## Sources of funding

No sponsor. Study was conducted at Brandon regional hospital.

## Ethical approval

The study has been exempted by our institution.

## Consent

Written informed consent was obtained from the patient for publication of this case report and accompanying images. A copy of the written consent is available for review by the Editor-in-Chief of this journal on request.

## Author contribution

Sahned Jaafar, MD was resident involved during the case and also writing the manuscript.

Suy sen Hung Fong, MD was resident involved during the case and also writing the manuscript.

Abdul Waheed, MD were involved in writing the manuscript and editing the draft of the manuscript.

Subhasis Misra, MD, MS, FACS edited the manuscript and supervised the manuscript development.

Keyur Chavda, MD was the senior surgeon; and also the guarantor of the study; who performed the case and approved the final version of the manuscript.

## Registration of research studies

N/A.

## Guarantor

Keyur Chavda, MD.

## Provenance and peer review

Not commissioned, externally peer-reviewed.

## References

[1] Rex D.K. (2015). Quality indicators for colonoscopy. Gastrointest. Endosc..

[2] Sovich J.L., Sartor Z., Misra S. (2015). Developments in screening tests and strategies for colorectal cancer. Biomed Res. Int..

[3] Ro T.H., Mathew M.A., Misra S. (2015). Value of screening endoscopy in evaluation of esophageal, gastric and colon cancers. World J. Gastroenterol..

[4] Joseph D.A. (2016). Colorectal cancer screening: estimated future colonoscopy need and current volume and capacity. Cancer.

[5] Tiwari A. (2017). Recognition of extraperitoneal colonic perforation following colonoscopy: a review of the literature. Case Rep. Gastroenterol..

[6] Lohsiriwat V. (2010). Colonoscopic perforation: incidence, risk factors, management and outcome. World J. Gastroenterol..

[7] Luning T.H. (2007). Colonoscopic perforations: a review of 30,366 patients. Surg. Endosc..

[8] Agha R.A., Borrelli M.R., Farwana R., Koshy K., Fowler A., Orgill D.P., For the SCARE Group (2018). The SCARE 2018 statement: updating consensus surgical case report (SCARE) guidelines. Int. J. Surg..

[9] Chen Tzu-Chun, Hung Ji-Shiang, Lin Been-Ren, Huang John, Liang Jin-Tung (2017). Long-term follow-up for patients with colonic perforation due to colonoscopy: from clinical and medicolegal viewpoints. Formos. J. Surg..

[10] CDC (2018). Colorectal Cancer Screening Capacity in the United States. https://www.cdc.gov/cancer/dcpc/research/articles/crc_screening_model.htm.

[11] Khan M. (2017). Post-colonoscopy colonic perforation presenting with subcutaneous emphysema: a case report. Gastroenterology Res..

[12] Wexner S.D., Garbus J.E., Singh J.J. (2001). A prospective analysis of 13,580 colonoscopies. Reevaluation of credentialing guidelines. Surg. Endosc..

[13] Garcia Martinez M.T. (2007). Perforation after colonoscopy: our 16-year experience. Rev. Esp. Enferm. Dig..

[14] Ho H.C. (1996). Colon perforation, bilateral pneumothoraces, pneumopericardium, pneumomediastinum, and subcutaneous emphysema complicating endoscopic polypectomy: anatomic and management considerations. Am. Surg..

[15] Dehal A., Tessier D.J. (2014). Intraperitoneal and extraperitoneal colonic perforation following diagnostic colonoscopy. JSLS.

[16] Daniels I.R., Sullivan T., Hale J. (1999). Retroperitoneal gas after colonoscopy. J. R. Soc. Med..

[17] Cai S.L. (2015). Management of iatrogenic colorectal perforation: from surgery to endoscopy. World J. Gastrointest. Endosc..

[18] Cho S.B. (2012). Therapeutic options for iatrogenic colon perforation: feasibility of endoscopic clip closure and predictors of the need for early surgery. Surg. Endosc..

[19] Tam M.S., Abbas M.A. (2013). Perforation following colorectal endoscopy: what happens beyond the endoscopy suite?. Perm. J..

